# Aging-Induced Stem Cell Mutations as Drivers for Disease and Cancer

**DOI:** 10.1016/j.stem.2015.05.002

**Published:** 2015-06-04

**Authors:** Peter D. Adams, Heinrich Jasper, K. Lenhard Rudolph

**Affiliations:** 1 University of Glasgow and Beatson Institute for Cancer Research, Glasgow G61 1BD, UK; 2 Buck Institute for Research on Aging, 8001 Redwood Boulevard, Novato, CA 94945-1400, USA; 3 Leibniz Institute for Age Research - Fritz Lipmann Institute e.V. (FLI), Beutenbergstr. 11, 07745 Jena, Germany

## Abstract

Aging is characterized by a decrease in genome integrity, impaired organ maintenance, and an increased risk of cancer, which coincide with clonal dominance of expanded mutant stem and progenitor cell populations in aging tissues, such as the intestinal epithelium, the hematopoietic system, and the male germline. Here we discuss possible explanations for age-associated increases in the initiation and/or progression of mutant stem/progenitor clones and highlight the roles of stem cell quiescence, replication-associated DNA damage, telomere shortening, epigenetic alterations, and metabolic challenges as determinants of stem cell mutations and clonal dominance in aging.

## Teaser

In this review, Rudolph and colleagues discuss age-associated increases in the initiation and/or progression of mutant stem/progenitor clones and the roles of stem cell quiescence, replication-associated DNA damage, telomere shortening, epigenetic alterations, and metabolic challenges as determinants of stem cell mutations and clonal dominance in aging.

## Main Text

### Introduction

The incidence of tissue dysfunction, diseases, and many types of cancer, including colorectal cancer (CRC) and some types of leukemia, exponentially increases with age, and aging represents the single biggest risk factor for most cancers (Smith et al., 2009; Yancik, 2005). However, the reasons for this aging-associated failure in tissue maintenance and the increase in cancer are poorly understood. Without a doubt, cancer is largely driven by genome dysfunction, frequently exemplified by specific genetic alterations that drive one or more specific cancer phenotypes (Hanahan and Weinberg, 2011). Overwhelming evidence indicates that the genesis and progression of cancer depend on accumulation of genetic alterations. This evidence includes epidemiological modeling data (Armitage and Doll, 1954), in vitro cell transformation studies (Hahn et al., 1999; Land et al., 1983), analysis of hereditary cancer predisposition syndromes (Knudson, 1971), molecular pathology of cancer progression (Kinzler and Vogelstein, 1996), and recent large-scale complete sequencing of cancer exomes and genomes (Alexandrov et al., 2013).

The decline in functional capacity and genetic integrity of adult tissue stem cells is thought to be a major factor in the decline in tissue maintenance and the increase in cancer formation during aging (Behrens et al., 2014; Rando, 2006). Linking the genetic and stem cell models of cancer, a recent study proposed that the accumulation of mutations through stem cell divisions is a major determinant of lifetime cancer risk (Tomasetti and Vogelstein, 2015). However, this model does not easily explain the exponential increase in cancer incidence with age, nor the recently discovered exponential increase in clonally expanded mutant stem cells in the male germline, the hematopoietic system, and the intestinal epithelium of aging humans (Busque et al., 2012; Genovese et al., 2014; Jaiswal et al., 2014; Greaves et al., 2006; Hsieh et al., 2013; Goriely and Wilkie, 2012).

### Clonal Dominance of Mutant Stem and Progenitor Cells Increases Exponentially with Age

Recent studies on stem and progenitor cells have begun to shed light on the exponential age-dependent increase in cancer. Aging is associated with an exponential increase in the occurrence of clonal hematopoiesis, where a single mutant hematopoietic stem or progenitor cell (HSPC) contributes to a significant, measurable clonal proportion of mature blood lineages (Busque et al., 2012; Genovese et al., 2014; Jacobs et al., 2012; Jaiswal et al., 2014; Laurie et al., 2012; Shlush et al., 2014; Xie et al., 2014). Evolution of mutant clonal hematopoiesis with age predicts leukemia risk and the risk of other aging-associated diseases (Genovese et al., 2014; Jaiswal et al., 2014; Shlush et al., 2014). Of note, most of the mutations that result in clonal hematopoiesis in aging humans are leukemia related and recurrently affect the same set of genes (Busque et al., 2012; Genovese et al., 2014; Jaiswal et al., 2014; Shlush et al., 2014; Xie et al., 2014). These data indicate that the mutations are non-neutral and strongly selected for in aging. Mutant clones can acquire additional mutations and the sequential evolution of clones with multiple mutations was observed in primary, secondary, and tertiary clones within the pre-malignant HSC compartment of acute myeloid leukemia (AML) patients (Jan et al., 2012). Deep-sequencing analysis of blood samples from large human cohorts detected mutant, clonal hematopoiesis in a low frequency (< 0.9%) of people below the age of 45 years. However, above the age of 45 years the frequency of mutant, clonal hematopoiesis rises greatly, affecting 25%–70% of people at the age of > 70 years, depending on the sensitivity of the method of detection (Genovese et al., 2014; Jaiswal et al., 2014; McKerrell et al., 2015).

Aging is also associated with clonal selection of aberrant intestinal stem cells (ISCs). The ISC compartment is divided into separate crypt units, each containing 7–14 SCs. Neutral drifts within each crypt lead to clonal dominance of single ISCs in about 3- to 8-month time intervals in mice and in time intervals of up to 8 years in humans (Kim and Shibata, 2002; Lopez-Garcia et al., 2010; Snippert et al., 2010). Although the number of analyzed individuals is low, clonal crypt-dominance of ISCs harboring chromosomal gains and losses or mitochondrial DNA mutations appears to accumulate during aging in the human intestinal epithelium (Greaves et al., 2006; Hsieh et al., 2013).

Taken together, several observations argue that the clonal expansion of mutant stem and progenitor cells does not follow a strictly linear kinetic over an individual’s lifespan. Rather, the clonal dominance of such mutations increases exponentially during aging.

For example, studies on the male germline identified a set of genetic diseases that display a significant parental age effect (PAE), in which an increased prevalence of PAE disease is observed in the offspring of older fathers (reviewed in Goriely and Wilkie, 2012; Maher et al., 2014). Studies on PAE diseases revealed that simple additive models of replication errors could not explain the exponential increases in disease incidence in offspring of older fathers. Instead, mutations in the Ras pathway were identified as the cause of PAE diseases leading to “selfish” expansion of mutant spermatogonial stem cells and exponential increases in the mutant clone size in the testis of aging men compared to spermatogonial stem cells carrying neutral mutations (Goriely and Wilkie, 2012). Whether aging itself promotes the clonal dominance of non-neutral mutations in spermatogonial stem cells remains to be addressed in experimental settings.

In the hematopoietic system, studies revealed age-associated increases in the incidence of base-pair mutations in HSPCs. Of note, some mutations are detectable early in life and show rather linear increases with age, while others (e.g., mutations in spliceosome factors) become detectable only late in life and exhibit exponential increases with aging (McKerrell et al., 2015). These data indicate that aging affects the clonal dominance of stem/progenitor cell mutations but the effect size appears to depend on cell intrinsic process, e.g., the type of mutation and the affected pathways. In addition to base pair mutations, there is evidence for an aging-associated increase in clonal mosaicism originating from HSPCs with large chromosomal anomalies (deletions, duplications, and acquired uniparental disomies). In humans, such lesions are detectable at a constantly low frequency of 0.2%–0.5% until the age of 50 years. Thereafter, this percentage rises rapidly to 2%–2.5% by the age of 80 years (Jacobs et al., 2012; Laurie et al., 2012). Similarly, twin studies revealed that mosaic anomalies were undetectable at a young age but sharply increased in pairs > 55 years old (Forsberg et al., 2012). Mosaic individuals show an increased cancer risk and recurrent anomalies affect important genes known to control incidence of hematopoietic cancers, such as TET2 and DNMT3a (Jacobs et al., 2012; Laurie et al., 2012). Together, these data suggest that cancer-associated genetic alterations are present at detectable levels in hematopoietic cells of some younger individuals but clonal dominance of these alterations apparently increases exponentially during aging, presumably through selection for advantaged clones. In line with this interpretation, X-inactivation markers in healthy women appear to be stable and do not show clonal expansion during aging (Swierczek et al., 2008). Presumably, such markers do not confer a selective advantage on clones.

Aging-associated increases in stem and progenitor cell mutations can in principle be driven by increases in mutation initiation rates and/or in the selection and clonal dominance of stem and progenitor cells that have acquired non-neutral mutations. The use of inducible in vivo lineage tracing is key to investigating the influence of age on mutation initiation and clonal selection. In the mouse intestine, such approaches have allowed the detection and tracing of mutation initiation and clonal dominance of labeled stem cells (Kozar et al., 2013; Ritsma et al., 2014; Snippert et al., 2010; Vermeulen et al., 2013). Continuous labeling confirmed that the rate of initiation of neutral mutations (loss of base pairs in a (CA)_30_ base pair-repeat tract leading to activation of a marker gene) and the clonal dominance of such neutral mutations does not change over the lifetime of mice (Kozar et al., 2013; Tao et al., 1993, Winton et al., 1988). Although these studies on the intestine did not reveal an influence of age on the induction of single-base-pair mutation per se, it should be noted that (1) the induction rate of other types of mutations, e.g., large chromosomal abnormalities, may not be constant during lifetime (see below discussion on telomeres as a cause of chromosomal instability) and (2) aging-induced increases in base-pair mutation may be restricted to specific tissue types (see below discussion on aging-induced impairments in replication in hematopoietic stem cells).

Inducible expression of a reporter, along with the induction of colorectal cancer (CRC) driver mutations, revealed that some CRC driver mutations (e.g., oncogenic Ras) promote clonal dominance of mutant ISCs compared to competing wild-type ISCs within the crypt under homeostatic conditions in young mice (Vermeulen et al., 2013). However, these studies also showed that bona fide CRC-driver mutations are often outcompeted by non-mutant ISCs. In addition, the clonal dominance of CRC-driver mutations can be context dependent. For example, deletion of p53 promoted clonal dominance of ISCs in the context of intestinal inflammation but not in the non-inflamed intestine (Vermeulen et al., 2013). Together, these studies indicate that clonal dominance of non-neutral (CRC driver) mutations occurs in the intestinal epithelium and this can be context dependent. The influence of the context of aging remains to be delineated.

**Figure 1:**
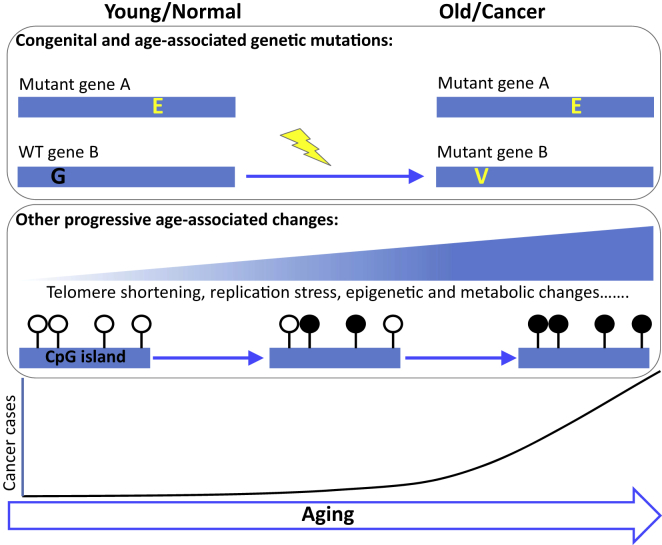
Multiple Factors Conspire to Drive Age-Associated Cancer Some congenital cancer-causing mutations are thought to be well-tolerated by young cells and tissues. Other cancer-causing mutations are acquired through aging. These congenital and acquired mutations conspire with other more progressive events, e.g., telomere shortening, replication stress, epigenetic and metabolic changes, to drive a dramatic increase in late-life cancer. Open lollipops, unmethylated CpG; filled lollipop, methylated CpG; yellow lightning bolt, genotoxic/mutagenic event.

Overall, where there is available experimental evidence, e.g., in the intestinal epithelium, it invariably points to linearity in de novo mutation rates with age. However, it was shown that chronic low doses of exogenous genotoxic stress caused exponential increases in mutation rates at the mouse *Dbl1* gene in the intestinal epithelium (Shaver-Walker et al., 1995). It remains to be investigated whether aging-associated cell-intrinsic increases in genotoxic stress, such as telomere shortening, replication stress, and epigenetic alterations (see below), could change mutation rates possibly in a tissue-specific manner. Along these lines, studies on human genetic diseases of impaired telomere maintenance revealed tissue specificity in regard to organ failure and cancer initiation, both being more pronounced in the hematopoietic system compared to the intestinal epithelium (Calado and Young 2009). In addition, aging appears to influence the kinetics of clonal selection of non-neutral, oncogenic mutations in somatic stem and progenitor cell compartments. Both processes—mutation initiation and their clonal selection—need to be investigated in a tissue-specific manner employing animal models that are relevant to human aging. Below we discuss molecular mechanisms that may increase somatic mutations during aging (Figure 1).

### Stem Cell Quiescence: Preserving Genome Integrity of Aging Stem Cells?

**Figure 2:**
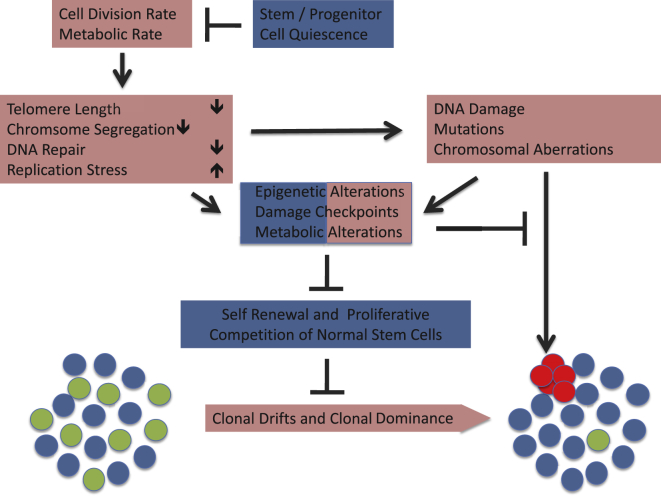
Aging-Induced Initiation and Clonal Selection of Stem and Progenitor Cell Mutations Aging-associated alterations that could contribute to the exponential increase in the initiation of DNA damage, mutations, and large chromosomal aberrations include telomere shortening and the dysregulation of components that control chromosome segregation, DNA replication, or DNA repair. There is evidence that cell division rates and metabolic activity induce these alterations, whereas stem and progenitor cell quiescence can prevent it. Genomic damages induce checkpoint responses, epigenetic alterations, and metabolic shifts that decrease the fitness of the damaged cells. These responses represent a double-edged sword. In young tissues, these responses are tumor protective by inhibiting the survival or the clonal expansion of single, individual stem and progenitor cells that acquire mutations or chromosomal aberrations. In contrast, the same responses can also limit the self-renewal and proliferative capacity of a growing number of normal stem and progenitor cells in aging tissues, which in turn promotes decreases in clone number, clonal drifts, and clonal dominance of mutant stem cells. Accordingly, the downregulation of damage responses/checkpoints is positively selected for both in the pool of aging stem and progenitor cells as well as in individual stem cells that acquire mutations or chromosomal aberrations. Red boxes define processes that contribute to the initiation and/or clonal dominance of mutant stem and progenitor cells, whereas processes depicted in blue boxes impair it. Epigenetic alterations, damage checkpoints, and metabolic alterations in response DNA damage and mutations have a dual role by affecting both the fitness of normal stem and progenitor cells as well as of mutants.

A small number of quiescent hematopoietic stem cells (HSCs) stand at the top of the hierarchy of the hematopoietic system, giving rise to all lineages of the peripheral blood. HSCs rarely divide in the course of several months (Wilson et al., 2008). This rarity of division of HSCs, also referred to as stem cell quiescence, has been shown to protect HSCs from accumulation of molecular damage, including the induction of replication-associated DNA damage, oxidative-stress-induced damages, and telomere shortening (Walter et al., 2015, Rossi et al., 2007, Choudhury et al., 2007) (Figure 2). There is evidence that the functionality of HSCs decreases with each round of division (Beerman et al., 2013) and aging-associated defects in DNA replication may aggravate these processes (Flach et al., 2014). Despite the evidence that quiescence protects HSCs from DNA damage accumulation and functional decline, there is also evidence that HSC quiescence promotes the accumulation of DNA damage and mutations by allowing the survival of damaged cells and error-prone repair (Mohrin et al., 2010, Beerman et al., 2014). However, cell-cycle entry of damaged HSCs promotes DNA repair or the removal of damaged HSCs (Beerman et al., 2014, Wang et al., 2014, Walter et al., 2015).

Together, there is clear evidence that quiescence protects HSCs from functional exhaustion, but the net effect of stem cell quiescence on initiation and clonal dominance of DNA mutations in the aging hematopoietic system remains to be studied in greater detail. In addition, the precise cell of origin for aging-associated, mutant, clonal hematopoiesis is not yet defined. Recent studies in mice revealed that under homeostatic conditions hematopoiesis is maintained predominantly by early hematopoietic progenitor cells or short-term HSCs but does not require a very active involvement of long-term HSCs (Busch et al., 2015; Sun et al., 2014b). However, in response to stress, long-term HSCs are activated to contribute to hemato-/lymphopoiesis (Busch et al., 2015). Of course, it remains to be seen to what extent hematopoietic homeostasis in laboratory mice kept under pathogen- and stress-free conditions mimics the situation in animals in the wild or in humans exposed to various types of stress during life, including fasting, infections, weather extremes, etc. Nevertheless, a significant contribution of progenitor cells and short-term HSCs to clonal expansion of mutant hematopoietic cells should not be excluded at this time, and future studies need to better define the cell type of origin of initiation and clonal dominance of mutant hematopoiesis. It should, however, be noted that maintenance of quiescence could also be relevant for mutation prevention at the early progenitor cell level, as there is clear evidence that early hematopoietic progenitor cells maintain a higher level of quiescence than downstream progenitors (Passegué et al., 2005). Accordingly, the maintenance of quiescence of stem and progenitor cells should be considered as an important, yet to be experimentally proven, cellular mechanism to prevent premature increases in DNA mutations leading to tissue aging and cancer formation. Experiments on animal models are required to determine candidate mechanisms that could influence the rate of somatic mutations in a replication/quiescence-dependent manner. Candidate mechanisms that could be involved include replication-associated errors and imperfect repair resulting in base mutation, or inter-chromosomal rearrangement induced for example by chromosomal instability and missegregation in response to replication-coupled telomere dysfunction (see below). Tomasetti and Vogelstein recently invoked replication-coupled stem cell mutations to explain the strong relationship between lifetime cancer risk and number of stem cell divisions across tissues (Tomasetti and Vogelstein, 2015). Extending this concept, there are many examples of chronic diseases that cause increased rates of cell turnover in the affected organs, which in turn associates with an increased risk of early cancer development in those organs (see for example connection between cirrhosis and liver cancer in chronic liver disease as reviewed in El-Serag and Rudolph, 2007).

Molecular mechanisms that control stem cell quiescence are emerging (Maryanovich et al., 2012; Sirin et al., 2010; Tsai et al., 2013; Walkley et al., 2005; Ye et al., 2013). Interestingly, several factors critical for the maintenance of HSC quiescence also influence health and lifespan. The transcription factor Nrf2, for example, has an evolutionarily conserved role in regulating stem cell quiescence, including of ISCs in *Drosophila* (Biteau et al., 2011) and of HSCs in mice (Tsai et al., 2013). Nrf2 has been implicated in the positive effect of calorie restriction on lifespan, which in mice is associated with a suppression of aging-associated cancer formation (Pearson et al., 2008). Other factors required for HSC quiescence include ATM, which has a known role in protecting cells and tissues from premature aging by promoting DNA repair and antioxidant defense (Maryanovich et al., 2012), and CEBP/a (Ye et al., 2013). Furthermore, loss of p21-dependent stem cell quiescence can lead to aging-associated stem cell depletion across various tissues in aging mice, including in HSCs (Cheng et al., 2000; Kippin et al., 2005).

A direct link between the age-related loss of stem cell quiescence and decline of tissue function and associated increase in mortality has been demonstrated in the intestinal epithelium in *Drosophila*. In young animals, the ISC population is largely quiescent but can be activated rapidly upon stress or tissue damage (Amcheslavsky et al., 2009; Biteau et al., 2008, 2011; Biteau et al., 2010; Buchon et al., 2009). The proliferative response of ISCs to damage is transient, and a return to the quiescent state after a regenerative episode is critical for tissue homeostasis (Biteau et al., 2010; Buchon et al., 2009). In old animals, this control of ISC proliferation is lost, resulting in epithelial dysplasia. Excessive ISC proliferation in aging intestines is caused by reactive oxygen species (ROS) that are produced by Dual Oxidase (Duox), an enzyme that generates an oxidative burst to control commensal and pathogenic bacteria. In old flies, Duox expression and activity is increased in the intestinal epithelium due to immune dysfunction and commensal dysbiosis (Biteau et al., 2008, 2010; Buchon et al., 2009; Guo et al., 2014). Promoting stem cell quiescence through various interventions, such as reducing stress-activated signaling pathways, improving immune homeostasis, limiting commensal dysbiosis, or reducing ISC proliferation directly, is sufficient to improve tissue homeostasis in the intestinal epithelium of old flies and extend lifespan (Biteau et al., 2010; Guo et al., 2014). While these studies highlight the importance of stem cell quiescence for tissue maintenance, it remains unclear whether age-related over-proliferation of *Drosophila* ISCs is associated with increased genomic alterations. However, lineage tracing has revealed defects in differentiation capacity of old ISCs, suggesting changes not only in activity, but also in functionality of these cells (Biteau et al., 2008). Whether these changes are driven by accumulated mutations, epigenetic changes, or extrinsic influences in the aging tissue remains to be established.

In contrast to the ISC system in *Drosophila*, the mouse intestine is maintained by highly proliferative LGR5^+^ ISCs dividing at approximately 24-hr intervals (Barker et al., 2007). Each intestinal basal crypt contains 7–14 ISCs competing against each other for self-renewal (de Navascués et al., 2012; Kozar et al., 2013; Snippert et al., 2010). A critical determinant of competitive advantage is the position of each ISC relative to the Wnt3-producing Paneth cells (see above, for review Clevers, 2013). In this population of highly proliferative ISCs, the maintenance of quiescence is likely not an important factor for maintenance of self-renewal, nor for preventing initiation and/or clonal dominance of mutations in these stem cells during aging. Interestingly, it was shown that the level of Wnt-signaling activity depending on the positioning of ISCs in the stem cell niche influences the survival of ISCs in the context of DNA damage. ISCs with low Wnt-activity preferentially survive compared to ISCs with high Wnt-activity in response to both telomere shortening and irradiation-induced DNA damage (Tao et al., 2015). These data suggest that niche positioning and Wnt-activity could influence the cell type of origin and the rate of mutation initiation in the context of DNA damage. In sum, it is likely that tissue context and differences in the stem cell biology across different tissues and species influence the requirement for stem cell quiescence for suppression of DNA damage, genetic mutations, and maintenance of stem cell function.

### Telomere Shortening Initiates Clonal Drift in Stem Cell Pools and Clonal Dominance of Mutant Stem Cells

Telomere shortening is a well-defined mechanism underpinning cell mortality, which could also contribute to exponential increases in genomic instability of adult cells in response to replicative aging, when critical telomere shortening leads to telomere uncapping and the induction of genomic instability. Studies from yeast to mammals provided experimental evidence for this interrelationship (Begus-Nahrmann et al., 2009; Hackett and Greider, 2003; Rudolph et al., 2001, Seluanov et al., 2004). Telomeres shorten in almost all human tissues during aging, including HSPCs (Vaziri et al., 1994) and the intestinal epithelium (Hiyama et al., 1996). These data indicate that telomerase activity—although required to ensure the long replicative lifespan of stem and progenitor cells (Holohan et al., 2014; Rudolph et al., 1999) —apparently is not sufficient to prevent age-associated shortening of telomeres in stem and progenitor cells. Moreover, accelerated shortening of telomeres in stem cell compartments of the intestinal epithelium and the hematopoietic system occurs in the context of chronic diseases and is linked to evolution of chromosomal instability, tissue dysfunction, and an increased cancer risk in humans (Risques et al., 2008; Townsley et al., 2014; Wang et al., 2012).

Telomere shortening may enhance initiation and evolution of clonal dominance of mutant stem cells during tissue aging (Figure 2). Critically short telomeres induce chromosomal instability and loss of heterozygosity in yeast (Hackett and Greider, 2003). Studies on telomerase-deficient mice revealed that, in the intestinal epithelium, telomere dysfunction induces clonal dominance of chromosomal unstable ISCs, albeit only in the context of p53 deficiency (Begus-Nahrmann et al., 2009). Of note, the increase in clonal dominance of chromosomal unstable ISCs resulted in accelerated tissue dysfunction and lifespan shortening of the mice, thus providing a proof of concept that stem cell mutations not only contribute to cancer development but can also aggravate aging-associated tissue dysfunction (Begus-Nahrmann et al., 2009).

In the hematopoietic system, telomere dysfunction accelerated aging-associated drifts within the pool of HSCs (Wang et al., 2012) (Figure 2). Typically, aging is characterized by a dominance of myeloid-biased HSPCs, preferentially differentiating into the myeloid lineage, and a paucity in lymphoid-biased HSPCs, preferentially differentiating into the lymphoid lineage (Ju et al., 2007; Wang et al., 2012). Studies showed that telomere dysfunction induced alteration in the stem cell environment, as well as a DNA-damage-dependent, differentiation-inducing checkpoint that promoted the preferential loss of self-renewing lymphoid-biased HSCs (Ju et al., 2007; Wang et al., 2012).

Together, studies in telomerase-deficient mice provided experimental evidence that telomere dysfunction promotes clonal drifts in the pool of tissue stem cells, as well the initiation and clonal dominance of mutant stem cells with large chromosomal abnormalities (Figure 2). The prevalence of telomere shortening in aging human tissues also suggests that telomere dysfunction could contribute to the observed increases in clonal dominance of chromosomal unstable stem cells in human intestinal epithelium (Hsieh et al., 2013) and the hematopoietic system (Jacobs et al., 2012; Laurie et al., 2012) in the context of aging. Despite experimental evidence on the role of telomere shortening in inducing chromosomal instability, it is surprising that there is an apparent lack of knowledge on the role of telomere shortening in promoting the initiation of gene mutations in stem and progenitor cell compartments in aging mammals. Interestingly, it was recently shown that telomere dysfunction induces defects in mRNA splicing by suppressing the expression of SRSF2 (Colla et al., 2015), which represents one of the genes affected by mutations that exhibit a strong positive selection during human aging (McKerrell et al., 2015). These data suggest that telomere shortening in the hematopoietic system promotes the clonal dominance of aging-associated stem and progenitor cell mutations.

In addition to telomere shortening, molecular components that regulate chromosome segregation, such as BubR1, influence tissue aging. Genetic experiments in mice showed that the downregulation of BubR1 induces aneuploidy and premature aging (Baker et al., 2004), whereas increases in BubR1 expression extended the tissue maintenance, health, and lifespan (Baker et al., 2013; North et al., 2014). Of note, BubR1 exhibits a significant downregulation in several tissues during aging (Baker et al., 2004) and alterations in the expression of mitotic genes appeared as a characteristic feature of human aging (Ly et al., 2000). It is conceivable that these alterations affect the functionality of both stem and progenitor cells (Figure 2). The impact of aging-associated alterations in the expression of mitosis/chromosome-regulating genes on initiation and clonal dominance of stem and progenitor cell mutations remains to be investigated, given the evidence that failures in chromosome segregation can induce loss of heterozygosity and cancer formation (for review see Ricke et al., 2008).

### Replication Stress Increases in Aging Stem Cells

Suppression of replication stress likely contributes to the protective effects of quiescence to counter age-dependent accumulation of genetic alterations. Emerging data indicate that DNA replication stress represents a molecular mechanism contributing to the exponential increase in genome aberrations in populations of aging stem cells (Flach et al., 2014; Walter et al., 2015). Despite their quiescence (see previous section), aged HSCs acquire a problem in DNA replication due to an aging-associated decrease in the expression of mini-chromosome maintenance (MCM) helicase components (Flach et al., 2014). This defect appears not to disturb hematopoiesis under homeostatic conditions, but suppresses the functionality of aged HSCs in response to hematopoietic stress. Of note, it was shown that replication stress and DNA damage are triggered by a burst of oxidative stress when quiescent HSCs are stimulated to enter the cell cycle in response to physiological stimuli, such as blood loss or infection-induced stimuli, such as interferon signaling (Walter et al., 2015). HSC replication stress in response to such stimuli led to severe bone marrow failure in mice with a deficient Fanconia anemia (FA) repair pathway, thus suggesting a resolution to the paradox whereby the FA pathway-deficient mouse models show no severe defects in hematopoiesis under homeostatic conditions but FA patients suffer from severe anemia and bone marrow failure. Whether impairments in DNA replication in response to aging contribute to an increased frequency of DNA mutations in normal aging mice and humans remains to be investigated (Figure 2). However, these data indicate that experimental conditions that mimic “physiological” replication stress of the hematopoietic system are relevant for modeling hematopoietic diseases and aging in humans, which may not be modeled by homeostatic hematopoiesis in laboratory mice housed under stress-free conditions. This should be considered when exploring the role of DNA replication stress in driving the acquisition and clonal dominance of mutant HSCs in aging.

Replication stress was recently shown to have dual antagonistic roles in suppression and initiation of CIN/aneuploidy depending on the functionality of checkpoints. Replication stress is induced in response to aneuploidy and CIN in primary human cells and in human cancer cells (Burrell et al., 2013; Meena et al., 2015). In primary human cells with intact checkpoints, aneuploidy induces replication stress at telomeres, which in turn leads to aneuploidy-induced senescence suppressing the proliferative capacity of aneuploid cells (Meena et al., 2015). Of note, endogenous expression of telomerase in murine HSCs was sufficient to alleviate aneuploidy-induced replication stress at telomeres and aneuploidy-induced senescence. These data suggest that telomerase suppression in somatic cells may prevent the propagation of whole chromosomal gains and losses by promoting replication stress at telomeres in response to aneuploidy and, hence, aneuploidy-induced senescence. While telomerase is clearly required to ensure the replicative potential and to suppress telomere dysfunction-induced CIN of aging stem and progenitor cells, this also appears to increase the risk of these cells to accumulate aneuploidy.

In contrast to primary cells with intact checkpoints, the induction of replication stress in response to CIN was shown to aggravate the CIN-phenotype in checkpoint-deficient, human cancer cells (Burrell et al., 2013). These data indicate that the initiation and clonal dominance of chromosomal unstable cells may depend on both the telomerase expression status and the functionality of DNA damage checkpoints. Of note, studies on γ-irradiated mice revealed a significant aging-associated blunting in the induction of p53 checkpoint activity in hematopoietic tissues (Feng et al., 2007). Together it is possible that endogenous expression of telomerase in stem cells coupled with aging-associated defects in DNA damage checkpoints and increase in replication stress promote the emergence of chromosomal unstable stem and progenitor cell clones characteristic of human aging (Hsieh et al., 2013; Jacobs et al., 2012; Laurie et al., 2012). Despite the evidence for a decrease in DNA damage checkpoint fidelity in aging tissues, mutations of p53 and RB are frequently found in mutant clonal hematopoiesis associated with aging (Laurie et al., 2012; Xie et al., 2014). These data indicate that the loss of these checkpoint genes is positively selected for in aging clonal hematopoiesis, possibly triggered by impaired HSC function induced by replication stress, telomere shortening, or other types of DNA damage (Figure 2).

In addition to its antagonistic role in protection/induction of large chromosomal aberrations, it is possible that replication stress would also influence the initiation and/or clonal dominance of base-pair mutations in aging stem and progenitor cells (Figure 2). This shift could further be promoted by aging-associated defects in components of DNA repair pathways (Figure 2). There is some evidence that efficiency of non-homologous end joining DNA repair decreases in human fibroblasts entering the stage of replicative senescence in culture (Seluanov et al., 2004). Whether aging-associated repair deficiencies occur in stem and progenitor cells in vivo remains to be addressed experimentally.

### Epigenetic Alterations Could Influence Clonal Dominance of Non-neutral Stem and Progenitor Cell Mutations

Aging is also associated with changes to the epigenome. Key early reports showed this in various tissues, including intestine and white blood cells (Berdasco and Esteller, 2012). In the intestine, this has been confirmed at the level of individual intestinal crypts (Kim et al., 2005). More recently, comprehensive studies of mouse HSCs also showed changes in DNA methylation with age, including a small net hypermethylation both globally and at CpG islands during normal aging, and a more pronounced global hypomethylation when additional excessive proliferation is enforced through transplantation (Beerman et al., 2013; Sun et al., 2014a). Underscoring the importance of epigenetic control of aging, several reports have functionally linked epigenetic control to aging and longevity (Dang et al., 2009; Greer et al., 2010).

Analyses of stem cells have begun to define age-associated changes that might underlie age-associated stem cell dysfunction. For example, a comparison of muscle stem cells from young and old mice showed that old stem cells exhibit elevated repressive H3K27me3 at repressed histone genes (Liu et al., 2013) and a de-repression of the epigenetic regulated cell-cycle inhibitor and senescence inducer p16/Ink4a (Sousa-Victor et al., 2014). The former may be a consequence of decreased proliferative potential of these aged stem cells (Marzluff et al., 2008) but is also expected to reinforce and exacerbate degenerative age-associated chromatin changes by restricting homeostatic nucleosome dynamics, so-called “chromostasis” (Rai et al., 2014). Aged mouse HSCs accumulate DNA methylation changes expected to promote expression of self-renewal genes and impair expression of differentiation genes, including lymphoid genes, likely contributing to the reported corresponding phenotypes in aged HSCs (Sun et al., 2014a). Underscoring the importance of such age-associated epigenetic changes, recent human studies have identified relatively small numbers of CpGs whose age-associated change in methylation status in multiple tissues correlates strongly with chronological age and biological age, the latter linked to predisposition to disease and mortality (Hannum et al., 2013; Horvath, 2013; Marioni et al., 2015; Weidner et al., 2014).

**Figure 3:**
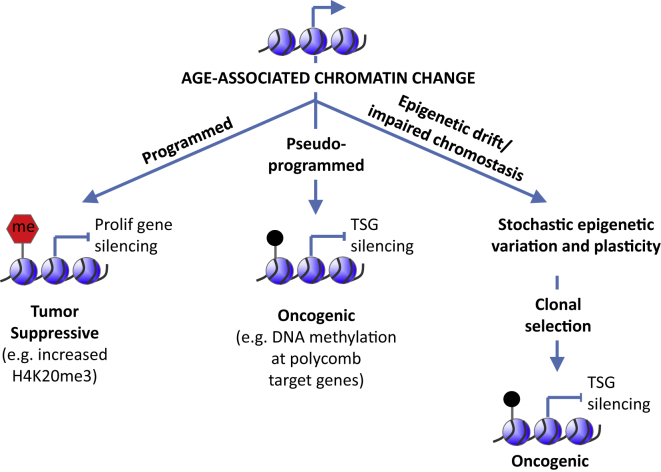
Age-Associated Chromatin Changes Exhibit Diverse Mechanisms, Functions, and Dysfunctions Some age-associated chromatin changes are tumor-suppressive programmed responses in stressed or damaged cells (left). Others are pseudo-programmed and in principle could be oncogenic (middle), tumor suppressive (not shown), or neutral (not shown). For example, bivalent gene promoters marked with H3K4me3 and H3K27me3 persist from ES cells into adult stem cells and facilitate developmental transitions. However, in adult tissues, bivalent promoters are, perhaps inadvertently, DNA methylated and silenced, thereby blocking differentiation. By this model, bivalent promoters are antagonistic pleiotropic—they facilitate developmental transitions in ES cells and young stem cells but can be precursors to self-renewing, non-differentiated cancer stem cells in old tissues. Other age-associated chromatin changes reflect epigenetic drift or failed chromostasis of the dynamic epigenome (right). Such drift is not inherently oncogenic or tumor suppressive but leads to stochastic epigenetic mosaicism and cell-to-cell variation, which can be a substrate for clonal selection of neoplastic cells.

Some age-associated epigenetic changes likely act as barriers to cell transformation and cancer (Figure 3). For example, histone modification H4K20me3 increases in aged tissues and senescent cells but is often downregulated in cancer (Berdasco and Esteller, 2012). Conversely, downregulation of H3K27me3 in aged pancreas is linked to derepression of tumor suppressor p16INK4a (Dhawan et al., 2009), and some cancers exhibit elevated H3K27me3 (Morgan and Shilatifard, 2015). Premature aged Hutchinson Gilford progeria cells are resistant to transformation, in part due to enhanced Brd4-mediated inhibition of oncogene-driven de-differentiation, linked to altered epigenetic programming (Fernandez et al., 2014). Such changes likely reflect programmed chromatin-mediated tumor suppressor mechanisms that are activated in stressed and/or aged cells.

Other age-associated chromatin changes likely promote cancer. Some such chromatin changes are, perhaps, best viewed as a form of epigenetic damage, in part due to stochastic drift of a dynamic epigenome (Figure 3). For example, like cancer, tissue aging has been reported to be associated with global DNA hypomethylation and more focal hypermethylation at CpG islands (Berdasco and Esteller, 2012). Similar results have been reported in senescent cells in culture (Cruickshanks et al., 2013). CpG islands methylated in aging and senescence include islands hypermethylated in cancer and thought to contribute to gene silencing (Berdasco and Esteller, 2012, Cruickshanks et al., 2013). In the hematopoietic system, some CpG islands show progressively increased methylation from young, to old, to myelodysplastic syndrome (MDS), and ultimately to AML (Maegawa et al., 2014). As well as acquiring epigenetic changes linked to increased self-renewal and decreased differentiation (see above), aged HSCs also display such hypermethylation events characteristic of MDS. Most notably, the gene *Sf3b1*, the human ortholog of which is mutated in about 20% of MDS, is methylated and underexpressed in aged mouse HSCs (Sun et al., 2014a). By contributing to silencing of such tumor suppressor genes, such age-associated methylation changes might predispose to cancer in aged cells. Poorly defined chromostatic mechanisms likely retard the development of such deleterious age-associated changes.

Paradoxically, however, some of these age-associated candidate oncogenic epigenetic changes might be pseudo-programmed within the epigenome itself (Figure 3). So-called bivalent genes marked with H3K4me3 and H3K27me3 in ES cells tend to be DNA methylated in aged tissues and methylated and silenced in cancer (Ohm et al., 2007; Rakyan et al., 2010; Schlesinger et al., 2007; Teschendorff et al., 2010; Widschwendter et al., 2007). Some adult stem cells, including hair follicle stem cells, muscle stem cells, and HSCs, also contain bivalent marked genes (Lien et al., 2011; Liu et al., 2013; Sun et al., 2014a). The polycomb (PRC) complex is responsible for deposition of H3K27me3, and, in HSCs, PRC target genes often succumb to age-related DNA methylation (Sun et al., 2014a). Rossi and coworkers proposed that, at least in some cases, DNA methylation results from downregulation of PRC complexes that otherwise antagonize DNA methylation (Beerman et al., 2013). Regardless of the mechanism, an epigenetic signature linked to bivalency in ES cells appears to track through adult stem cell populations and is, perhaps inadvertently, predisposed to methylation and stable gene silencing in aged cells. By this model, bivalent chromatin can be considered to be antagonistic pleiotropic (Williams, 1957)—advantageous in ES cells and young adult stem cells, but disadvantageous in old adult stem cells.

Cancer-causing genetic mutations in epigenetic regulators have been revealing regarding the tumor-suppressive functions of the normal epigenome and possible tumor-promoting roles of an altered aged epigenome. Mutations in epigenetic regulators, for example DNMT3a, TET2, and ASXL1, are frequently found in myeloid neoplasia (Shih et al., 2012). In a clutch of seminal papers, mutations in these epigenetic regulators have been revealed as the earliest genetic changes in the neoplastic progression. These mutations are associated with clonal dominance of HSCs that are, at least in the case of those harboring hypomorphic DNMT3a mutations, phenotypically normal, albeit at increased risk of myeloid malignancy (Corces-Zimmerman et al., 2014; Genovese et al., 2014; Jaiswal et al., 2014; Jan et al., 2012; Shlush et al., 2014; Xie et al., 2014). In mice, biallelic knockout of DNMT3a confers increased self-renewal and impaired differentiation of HSCs and predisposition to hematologic neoplasia, including MDS and AML (Mayle et al., 2015). In part, this reflects a role for DNMT3a in methylation and associated silencing of self-renewal genes, including Runx1 and β-catenin (Challen et al., 2012, 2014). However, inactivation of DNMT3a triggers other, apparently secondary, epigenetic changes, including DNMT3b-mediated hypermethylation and gene repression, and changes in histone modifications also linked to gene repression (Challen et al., 2012, 2014). Conceivably, as well as contributing to a programmed pro-differentiation/anti-self-renewal tumor suppressor function, DNMT3a might also promote chromostasis to restrict more stochastic epigenetic variation and plasticity in HSCs. While not directly tumor promoting, increased cell-to-cell variation and mosaicism resulting from inactivation of DNMT3a might be a substrate for clonal selection of rare pre-neoplastic cells (Figure 3). Some similarities in epigenetic changes due to DNMT3a inactivation and aging, for example in DNA methylation poor “canyons” (Challen et al., 2014; Sun et al., 2014a), suggest that inactivation of DNMT3a might, to some extent, accelerate the epigenetic drift and HSC mosaicism associated with normal aging and resultant predisposition to myeloid neoplasia. In turn, such epigenetic changes are likely to influence the selection for and against other genetic mutations, the response to replication stress and dysfunctional telomeres.

### Metabolism Influences Aging and Functionality of Stem Cells

Metabolism and signaling pathways that influence metabolism are major determinants of the rate of aging and longevity and also impinge on cancer. Studies in the nematode *C. elegans* provided the first experimental evidence that the reduction of Insulin-IGF1 signaling (IIS) increases lifespan (Kimura et al., 1997; Kenyon et al., 1992). Improvements in healthspan have since been demonstrated in response to dietary, small molecule, and genetic interventions in metabolism and associated signaling pathways (including IIS, mTORC1, and AMPK signaling) in some strains of laboratory mice and non-human primates (Kenyon, 2010). Genetic studies on centenarians further imply a role of the IIS pathway on longevity in humans (Flachsbart et al., 2009; Willcox et al., 2008). Importantly, metabolic interventions that increase longevity in mice tend to reduce the incidence of cancer (Serrano and Blasco, 2007). For example, inhibition of mTORC1 extends healthspan and longevity (Harrison et al., 2009), and a recent study has shown that inhibition of mTORC1 also suppresses intestinal tumor initiation and progression in APC deficient mice (Faller et al., 2015). mTORC1 inhibition benefits the maintenance of ISC function, either through non-autonomous effects from niche (Paneth) cells as described in the mouse intestine (Yilmaz et al., 2012), or through cell-autonomous prevention of ISC differentiation in the fly intestine (Kapuria et al., 2012).

However, the effects of these pathways are complex and diverse, and the mechanisms underlying the tumor-suppressive effects of reduced IIS and other metabolic signals remain to be determined. Studies on calorie restriction and fasting-mediated reduction in IIS revealed beneficial effects for the maintenance of stem cell function under homeostatic conditions and in response to stress (Cerletti et al., 2012; Cheng et al., 2014). Moreover, protective metabolic pathways (such as autophagy) were induced and required for stem cell maintenance in response to calorie restriction and may contribute to an improved clearance of damaged molecules and cells in the pool of aging stem cells (Warr et al., 2013). Long-term effects of such interventions on stem cell function remain yet to be investigated.

It is also conceivable that some of the beneficial effects of metabolic interventions involve suppression of cell-cycle activity of stem cells. Increased stem cell quiescence in response to such interventions may in turn delay the accumulation of aging-associated mutations and/or the clonal dominance of mutant stem cells in aging tissues (see above discussion on stem cell quiescence, replication stress, and tissue aging). Indeed, in the fly intestinal epithelium, ISC proliferation is regulated by both local and systemic insulin-like peptides (Amcheslavsky et al., 2009; Biteau et al., 2010; O’Brien et al., 2011), and modulating insulin signaling activity as well as target genes of Foxo is sufficient to limit over-proliferation of ISCs, promote tissue homeostasis, and extend lifespan (Biteau et al., 2010). Similarly, ISC homeostasis can be maintained by overexpressing the mitochondrial regulator PGC1a in ISCs specifically, resulting in increased tissue integrity in aging flies and extending lifespan (Rera et al., 2011).

In contrast to the beneficial effects of lowering IIS-signaling on aging, there is emerging evidence that metabolic pathways change in aging cells and tissues. Telomere dysfunction was shown to trigger metabolic changes, including impaired mitochondrial biogenesis, which was shown to aggravate defects on stem cell function and organ maintenance during aging (Missios et al., 2014; Passos et al., 2010; Sahin and DePinho, 2012). Mitochondrial dysfunction is among the best-investigated metabolic alterations that occur during aging in various tissues (Bratic and Larsson, 2013). Mutations in mitochondrial DNA can aggravate SC and tissue aging in mice (Kujoth et al., 2005; Norddahl et al., 2011; Trifunovic et al., 2004). In addition, there is emerging evidence that oncogene-induced senescence can increase the rate of glucose metabolism both in glycolytic and in oxidative pathways (Dörr et al., 2013; Kaplon et al., 2013). If transferrable to telomere dysfunction-induced senescence and tissue aging, increases in energy demand may occur in aging, possibly requiring a different diet at advanced ages as compared to adulthood. Along these lines, Igf1 and glucose application exhibited beneficial effects on tissue maintenance in mouse models of premature aging (Mariño et al., 2010; Missios et al., 2014). Regardless of the specific mechanisms and pathways, age-dependent metabolic shifts might influence mutation frequency, progression to clonal dominance, and cancer.

### Outlook

There is emerging evidence that aging induces changes in molecular pathways that accelerate the initiation and/or clonal dominance of mutations in stem and progenitor cells. The tight connection between aging-associated accumulation of stem and progenitor cell mutations with the failure of tissue maintenance and cancer suppression indicates a causal relationship between these factors. In addition to the cell-intrinsic mechanisms discussed here, there is increasing evidence that cell-extrinsic factors affect stem cell maintenance and possibly the selection of mutant stem and progenitor cells during aging. Likely, and potentially exciting, extrinsic candidates include aging-associated defects in the stem cell niches (Vas et al., 2012), alterations in the systemic/blood circulatory environment (Ju et al., 2007), changes in proliferative competition among stem and progenitor cells (Bilousova et al., 2005; Henry et al., 2010), inflammatory responses (Velarde et al., 2013), and defects in immune surveillance of damaged cells (Kang et al., 2011). The delineation of this interplay of cellular and molecular mechanisms that contribute to the initiation and selection of stem and progenitor cell mutations in the context of aging will undoubtedly help the development of therapies aiming to improve early detection, prevention, and risk assessment of aging-associated diseases, organ dysfunction, and cancer.

## References

[bib1] Alexandrov L.B., Nik-Zainal S., Wedge D.C., Aparicio S.A., Behjati S., Biankin A.V., Bignell G.R., Bolli N., Borg A., Børresen-Dale A.L., Australian Pancreatic Cancer Genome Initiative, ICGC Breast Cancer Consortium, ICGC MMML-Seq Consortium, ICGC PedBrain (2013). Signatures of mutational processes in human cancer. Nature.

[bib2] Amcheslavsky A., Jiang J., Ip Y.T. (2009). Tissue damage-induced intestinal stem cell division in Drosophila. Cell Stem Cell.

[bib3] Armitage P., Doll R. (1954). The age distribution of cancer and a multi-stage theory of carcinogenesis. Br. J. Cancer.

[bib4] Baker D.J., Jeganathan K.B., Cameron J.D., Thompson M., Juneja S., Kopecka A., Kumar R., Jenkins R.B., de Groen P.C., Roche P., van Deursen J.M. (2004). BubR1 insufficiency causes early onset of aging-associated phenotypes and infertility in mice. Nat. Genet..

[bib5] Baker D.J., Dawlaty M.M., Wijshake T., Jeganathan K.B., Malureanu L., van Ree J.H., Crespo-Diaz R., Reyes S., Seaburg L., Shapiro V. (2013). Increased expression of BubR1 protects against aneuploidy and cancer and extends healthy lifespan. Nat. Cell Biol..

[bib6] Barker N., van Es J.H., Kuipers J., Kujala P., van den Born M., Cozijnsen M., Haegebarth A., Korving J., Begthel H., Peters P.J., Clevers H. (2007). Identification of stem cells in small intestine and colon by marker gene Lgr5. Nature.

[bib7] Beerman I., Bock C., Garrison B.S., Smith Z.D., Gu H., Meissner A., Rossi D.J. (2013). Proliferation-dependent alterations of the DNA methylation landscape underlie hematopoietic stem cell aging. Cell Stem Cell.

[bib8] Beerman I., Seita J., Inlay M.A., Weissman I.L., Rossi D.J. (2014). Quiescent hematopoietic stem cells accumulate DNA damage during aging that is repaired upon entry into cell cycle. Cell Stem Cell.

[bib9] Begus-Nahrmann Y., Lechel A., Obenauf A.C., Nalapareddy K., Peit E., Hoffmann E., Schlaudraff F., Liss B., Schirmacher P., Kestler H. (2009). p53 deletion impairs clearance of chromosomal-instable stem cells in aging telomere-dysfunctional mice. Nat. Genet..

[bib10] Behrens A., van Deursen J.M., Rudolph K.L., Schumacher B. (2014). Impact of genomic damage and ageing on stem cell function. Nat. Cell Biol..

[bib11] Berdasco M., Esteller M. (2012). Hot topics in epigenetic mechanisms of aging: 2011. Aging Cell.

[bib12] Bilousova G., Marusyk A., Porter C.C., Cardiff R.D., DeGregori J. (2005). Impaired DNA replication within progenitor cell pools promotes leukemogenesis. PLoS Biol..

[bib13] Biteau B., Hochmuth C.E., Jasper H. (2008). JNK activity in somatic stem cells causes loss of tissue homeostasis in the aging Drosophila gut. Cell Stem Cell.

[bib14] Biteau B., Karpac J., Supoyo S., Degennaro M., Lehmann R., Jasper H. (2010). Lifespan extension by preserving proliferative homeostasis in Drosophila. PLoS Genet..

[bib15] Biteau B., Hochmuth C.E., Jasper H. (2011). Maintaining tissue homeostasis: dynamic control of somatic stem cell activity. Cell Stem Cell.

[bib16] Bratic A., Larsson N.G. (2013). The role of mitochondria in aging. J. Clin. Invest..

[bib17] Buchon N., Broderick N.A., Chakrabarti S., Lemaitre B. (2009). Invasive and indigenous microbiota impact intestinal stem cell activity through multiple pathways in Drosophila. Genes Dev..

[bib18] Burrell R.A., McClelland S.E., Endesfelder D., Groth P., Weller M.C., Shaikh N., Domingo E., Kanu N., Dewhurst S.M., Gronroos E. (2013). Replication stress links structural and numerical cancer chromosomal instability. Nature.

[bib19] Busch K., Klapproth K., Barile M., Flossdorf M., Holland-Letz T., Schlenner S.M., Reth M., Höfer T., Rodewald H.R. (2015). Fundamental properties of unperturbed haematopoiesis from stem cells in vivo. Nature.

[bib20] Busque L., Patel J.P., Figueroa M.E., Vasanthakumar A., Provost S., Hamilou Z., Mollica L., Li J., Viale A., Heguy A. (2012). Recurrent somatic TET2 mutations in normal elderly individuals with clonal hematopoiesis. Nat. Genet..

[bib21] Calado R.T., Young N.S. (2009). Telomere diseases. N. Engl. J. Med..

[bib22] Cerletti M., Jang Y.C., Finley L.W., Haigis M.C., Wagers A.J. (2012). Short-term calorie restriction enhances skeletal muscle stem cell function. Cell Stem Cell.

[bib23] Challen G.A., Sun D., Jeong M., Luo M., Jelinek J., Berg J.S., Bock C., Vasanthakumar A., Gu H., Xi Y. (2012). Dnmt3a is essential for hematopoietic stem cell differentiation. Nat. Genet..

[bib24] Challen G.A., Sun D., Mayle A., Jeong M., Luo M., Rodriguez B., Mallaney C., Celik H., Yang L., Xia Z. (2014). Dnmt3a and Dnmt3b have overlapping and distinct functions in hematopoietic stem cells. Cell Stem Cell.

[bib25] Cheng T., Rodrigues N., Shen H., Yang Y., Dombkowski D., Sykes M., Scadden D.T. (2000). Hematopoietic stem cell quiescence maintained by p21cip1/waf1. Science.

[bib26] Cheng C.W., Adams G.B., Perin L., Wei M., Zhou X., Lam B.S., Da Sacco S., Mirisola M., Quinn D.I., Dorff T.B. (2014). Prolonged fasting reduces IGF-1/PKA to promote hematopoietic-stem-cell-based regeneration and reverse immunosuppression. Cell Stem Cell.

[bib27] Choudhury A.R., Ju Z., Djojosubroto M.W., Schienke A., Lechel A., Schaetzlein S., Jiang H., Stepczynska A., Wang C., Buer J. (2007). Cdkn1a deletion improves stem cell function and lifespan of mice with dysfunctional telomeres without accelerating cancer formation. Nat. Genet..

[bib28] Clevers H. (2013). The intestinal crypt, a prototype stem cell compartment. Cell.

[bib29] Colla S., Ong D.S., Ogoti Y., Marchesini M., Mistry N.A., Clise-Dwyer K., Ang S.A., Storti P., Viale A., Giuliani N. (2015). Telomere dysfunction drives aberrant hematopoietic differentiation and myelodysplastic syndrome. Cancer Cell.

[bib30] Corces-Zimmerman M.R., Hong W.J., Weissman I.L., Medeiros B.C., Majeti R. (2014). Preleukemic mutations in human acute myeloid leukemia affect epigenetic regulators and persist in remission. Proc. Natl. Acad. Sci. USA.

[bib31] Cruickshanks H.A., McBryan T., Nelson D.M., Vanderkraats N.D., Shah P.P., van Tuyn J., Singh Rai T., Brock C., Donahue G., Dunican D.S. (2013). Senescent cells harbour features of the cancer epigenome. Nat. Cell Biol..

[bib32] Dang W., Steffen K.K., Perry R., Dorsey J.A., Johnson F.B., Shilatifard A., Kaeberlein M., Kennedy B.K., Berger S.L. (2009). Histone H4 lysine 16 acetylation regulates cellular lifespan. Nature.

[bib33] de Navascués J., Perdigoto C.N., Bian Y., Schneider M.H., Bardin A.J., Martínez-Arias A., Simons B.D. (2012). Drosophila midgut homeostasis involves neutral competition between symmetrically dividing intestinal stem cells. EMBO J..

[bib34] Dhawan S., Tschen S.I., Bhushan A. (2009). Bmi-1 regulates the Ink4a/Arf locus to control pancreatic beta-cell proliferation. Genes Dev..

[bib35] Dörr J.R., Yu Y., Milanovic M., Beuster G., Zasada C., Däbritz J.H., Lisec J., Lenze D., Gerhardt A., Schleicher K. (2013). Synthetic lethal metabolic targeting of cellular senescence in cancer therapy. Nature.

[bib36] El-Serag H.B., Rudolph K.L. (2007). Hepatocellular carcinoma: epidemiology and molecular carcinogenesis. Gastroenterology.

[bib37] Faller W.J., Jackson T.J., Knight J.R., Ridgway R.A., Jamieson T., Karim S.A., Jones C., Radulescu S., Huels D.J., Myant K.B. (2015). mTORC1-mediated translational elongation limits intestinal tumour initiation and growth. Nature.

[bib38] Feng Z., Hu W., Teresky A.K., Hernando E., Cordon-Cardo C., Levine A.J. (2007). Declining p53 function in the aging process: a possible mechanism for the increased tumor incidence in older populations. Proc. Natl. Acad. Sci. USA.

[bib39] Fernandez P., Scaffidi P., Markert E., Lee J.H., Rane S., Misteli T. (2014). Transformation resistance in a premature aging disorder identifies a tumor-protective function of BRD4. Cell Rep..

[bib40] Flach J., Bakker S.T., Mohrin M., Conroy P.C., Pietras E.M., Reynaud D., Alvarez S., Diolaiti M.E., Ugarte F., Forsberg E.C. (2014). Replication stress is a potent driver of functional decline in ageing haematopoietic stem cells. Nature.

[bib41] Flachsbart F., Caliebe A., Kleindorp R., Blanché H., von Eller-Eberstein H., Nikolaus S., Schreiber S., Nebel A. (2009). Association of FOXO3A variation with human longevity confirmed in German centenarians. Proc. Natl. Acad. Sci. USA.

[bib42] Forsberg L.A., Rasi C., Razzaghian H.R., Pakalapati G., Waite L., Thilbeault K.S., Ronowicz A., Wineinger N.E., Tiwari H.K., Boomsma D. (2012). Age-related somatic structural changes in the nuclear genome of human blood cells. Am. J. Hum. Genet..

[bib43] Genovese G., Kähler A.K., Handsaker R.E., Lindberg J., Rose S.A., Bakhoum S.F., Chambert K., Mick E., Neale B.M., Fromer M. (2014). Clonal hematopoiesis and blood-cancer risk inferred from blood DNA sequence. N. Engl. J. Med..

[bib44] Goriely A., Wilkie A.O. (2012). Paternal age effect mutations and selfish spermatogonial selection: causes and consequences for human disease. Am. J. Hum. Genet..

[bib45] Greaves L.C., Preston S.L., Tadrous P.J., Taylor R.W., Barron M.J., Oukrif D., Leedham S.J., Deheragoda M., Sasieni P., Novelli M.R. (2006). Mitochondrial DNA mutations are established in human colonic stem cells, and mutated clones expand by crypt fission. Proc. Natl. Acad. Sci. USA.

[bib46] Greer E.L., Maures T.J., Hauswirth A.G., Green E.M., Leeman D.S., Maro G.S., Han S., Banko M.R., Gozani O., Brunet A. (2010). Members of the H3K4 trimethylation complex regulate lifespan in a germline-dependent manner in C. elegans. Nature.

[bib47] Guo L., Karpac J., Tran S.L., Jasper H. (2014). PGRP-SC2 promotes gut immune homeostasis to limit commensal dysbiosis and extend lifespan. Cell.

[bib48] Hackett J.A., Greider C.W. (2003). End resection initiates genomic instability in the absence of telomerase. Mol. Cell Biol..

[bib49] Hahn W.C., Counter C.M., Lundberg A.S., Beijersbergen R.L., Brooks M.W., Weinberg R.A. (1999). Creation of human tumour cells with defined genetic elements. Nature.

[bib50] Hanahan D., Weinberg R.A. (2011). Hallmarks of cancer: the next generation. Cell.

[bib51] Hannum G., Guinney J., Zhao L., Zhang L., Hughes G., Sadda S., Klotzle B., Bibikova M., Fan J.B., Gao Y. (2013). Genome-wide methylation profiles reveal quantitative views of human aging rates. Mol. Cell.

[bib52] Harrison D.E., Strong R., Sharp Z.D., Nelson J.F., Astle C.M., Flurkey K., Nadon N.L., Wilkinson J.E., Frenkel K., Carter C.S. (2009). Rapamycin fed late in life extends lifespan in genetically heterogeneous mice. Nature.

[bib53] Henry C.J., Marusyk A., Zaberezhnyy V., Adane B., DeGregori J. (2010). Declining lymphoid progenitor fitness promotes aging-associated leukemogenesis. Proc. Natl. Acad. Sci. USA.

[bib54] Hiyama E., Tatsumoto N., Kodama T., Hiyama K., Shay J., Yokoyama T. (1996). Telomerase activity in human intestine. Int. J. Oncol..

[bib55] Holohan B., Wright W.E., Shay J.W. (2014). Cell biology of disease: Telomeropathies: an emerging spectrum disorder. J. Cell Biol..

[bib56] Horvath S. (2013). DNA methylation age of human tissues and cell types. Genome Biol..

[bib57] Hsieh J.C., Van Den Berg D., Kang H., Hsieh C.L., Lieber M.R. (2013). Large chromosome deletions, duplications, and gene conversion events accumulate with age in normal human colon crypts. Aging Cell.

[bib58] Jacobs K.B., Yeager M., Zhou W., Wacholder S., Wang Z., Rodriguez-Santiago B., Hutchinson A., Deng X., Liu C., Horner M.J. (2012). Detectable clonal mosaicism and its relationship to aging and cancer. Nat. Genet..

[bib59] Jaiswal S., Fontanillas P., Flannick J., Manning A., Grauman P.V., Mar B.G., Lindsley R.C., Mermel C.H., Burtt N., Chavez A. (2014). Age-related clonal hematopoiesis associated with adverse outcomes. N. Engl. J. Med..

[bib60] Jan M., Snyder T.M., Corces-Zimmerman M.R., Vyas P., Weissman I.L., Quake S.R., Majeti R. (2012). Clonal evolution of preleukemic hematopoietic stem cells precedes human acute myeloid leukemia. Sci. Transl. Med..

[bib61] Ju Z., Jiang H., Jaworski M., Rathinam C., Gompf A., Klein C., Trumpp A., Rudolph K.L. (2007). Telomere dysfunction induces environmental alterations limiting hematopoietic stem cell function and engraftment. Nat. Med..

[bib62] Kang T.W., Yevsa T., Woller N., Hoenicke L., Wuestefeld T., Dauch D., Hohmeyer A., Gereke M., Rudalska R., Potapova A. (2011). Senescence surveillance of pre-malignant hepatocytes limits liver cancer development. Nature.

[bib63] Kaplon J., Zheng L., Meissl K., Chaneton B., Selivanov V.A., Mackay G., van der Burg S.H., Verdegaal E.M., Cascante M., Shlomi T. (2013). A key role for mitochondrial gatekeeper pyruvate dehydrogenase in oncogene-induced senescence. Nature.

[bib64] Kapuria S., Karpac J., Biteau B., Hwangbo D., Jasper H. (2012). Notch-mediated suppression of TSC2 expression regulates cell differentiation in the Drosophila intestinal stem cell lineage. PLoS Genet..

[bib149] Kenyon C., Chang J., Gensch E., Rudner A., Tabtiang R. (1993). A C. elegans mutant that lives twice as long as wild type. Nature.

[bib65] Kenyon C.J. (2010). The genetics of ageing. Nature.

[bib66] Kim K.M., Shibata D. (2002). Methylation reveals a niche: stem cell succession in human colon crypts. Oncogene.

[bib67] Kim J.Y., Siegmund K.D., Tavaré S., Shibata D. (2005). Age-related human small intestine methylation: evidence for stem cell niches. BMC Med..

[bib68] Kimura K.D., Tissenbaum H.A., Liu Y., Ruvkun G. (1997). daf-2, an insulin receptor-like gene that regulates longevity and diapause in Caenorhabditis elegans. Science.

[bib69] Kinzler K.W., Vogelstein B. (1996). Lessons from hereditary colorectal cancer. Cell.

[bib70] Kippin T.E., Martens D.J., van der Kooy D. (2005). p21 loss compromises the relative quiescence of forebrain stem cell proliferation leading to exhaustion of their proliferation capacity. Genes Dev..

[bib71] Knudson A.G. (1971). Mutation and cancer: statistical study of retinoblastoma. Proc. Natl. Acad. Sci. USA.

[bib72] Kozar S., Morrissey E., Nicholson A.M., van der Heijden M., Zecchini H.I., Kemp R., Tavaré S., Vermeulen L., Winton D.J. (2013). Continuous clonal labeling reveals small numbers of functional stem cells in intestinal crypts and adenomas. Cell Stem Cell.

[bib73] Kujoth G.C., Hiona A., Pugh T.D., Someya S., Panzer K., Wohlgemuth S.E., Hofer T., Seo A.Y., Sullivan R., Jobling W.A. (2005). Mitochondrial DNA mutations, oxidative stress, and apoptosis in mammalian aging. Science.

[bib74] Land H., Parada L.F., Weinberg R.A. (1983). Tumorigenic conversion of primary embryo fibroblasts requires at least two cooperating oncogenes. Nature.

[bib75] Laurie C.C., Laurie C.A., Rice K., Doheny K.F., Zelnick L.R., McHugh C.P., Ling H., Hetrick K.N., Pugh E.W., Amos C. (2012). Detectable clonal mosaicism from birth to old age and its relationship to cancer. Nat. Genet..

[bib76] Lien W.H., Guo X., Polak L., Lawton L.N., Young R.A., Zheng D., Fuchs E. (2011). Genome-wide maps of histone modifications unwind in vivo chromatin states of the hair follicle lineage. Cell Stem Cell.

[bib77] Liu L., Cheung T.H., Charville G.W., Hurgo B.M., Leavitt T., Shih J., Brunet A., Rando T.A. (2013). Chromatin modifications as determinants of muscle stem cell quiescence and chronological aging. Cell Rep..

[bib78] Lopez-Garcia C., Klein A.M., Simons B.D., Winton D.J. (2010). Intestinal stem cell replacement follows a pattern of neutral drift. Science.

[bib79] Ly D.H., Lockhart D.J., Lerner R.A., Schultz P.G. (2000). Mitotic misregulation and human aging. Science.

[bib80] Maegawa S., Gough S.M., Watanabe-Okochi N., Lu Y., Zhang N., Castoro R.J., Estecio M.R., Jelinek J., Liang S., Kitamura T. (2014). Age-related epigenetic drift in the pathogenesis of MDS and AML. Genome Res..

[bib81] Maher G.J., Goriely A., Wilkie A.O. (2014). Cellular evidence for selfish spermatogonial selection in aged human testes. Andrology.

[bib82] Mariño G., Ugalde A.P., Fernández A.F., Osorio F.G., Fueyo A., Freije J.M., López-Otín C. (2010). Insulin-like growth factor 1 treatment extends longevity in a mouse model of human premature aging by restoring somatotroph axis function. Proc. Natl. Acad. Sci. USA.

[bib83] Marioni R.E., Shah S., McRae A.F., Chen B.H., Colicino E., Harris S.E., Gibson J., Henders A.K., Redmond P., Cox S.R. (2015). DNA methylation age of blood predicts all-cause mortality in later life. Genome Biol..

[bib84] Maryanovich M., Oberkovitz G., Niv H., Vorobiyov L., Zaltsman Y., Brenner O., Lapidot T., Jung S., Gross A. (2012). The ATM-BID pathway regulates quiescence and survival of haematopoietic stem cells. Nat. Cell Biol..

[bib85] Marzluff W.F., Wagner E.J., Duronio R.J. (2008). Metabolism and regulation of canonical histone mRNAs: life without a poly(A) tail. Nat. Rev. Genet..

[bib86] Mayle A., Yang L., Rodriguez B., Zhou T., Chang E., Curry C.V., Challen G.A., Li W., Wheeler D., Rebel V.I., Goodell M.A. (2015). Dnmt3a loss predisposes murine hematopoietic stem cells to malignant transformation. Blood.

[bib87] McKerrell T., Park N., Moreno T., Grove C.S., Ponstingl H., Stephens J., Crawley C., Craig J., Scott M.A., Hodkinson C., Understanding Society Scientific Group (2015). Leukemia-associated somatic mutations drive distinct patterns of age-related clonal hemopoiesis. Cell Rep..

[bib88] Meena J.K., Cerutti A., Beichler C., Morita Y., Bruhn C., Kumar M., Kraus J.M., Speicher M.R., Wang Z.Q., Kestler H.A. (2015). Telomerase abrogates aneuploidy-induced telomere replication stress, senescence and cell depletion. EMBO J..

[bib89] Missios P., Zhou Y., Guachalla L.M., von Figura G., Wegner A., Chakkarappan S.R., Binz T., Gompf A., Hartleben G., Burkhalter M.D. (2014). Glucose substitution prolongs maintenance of energy homeostasis and lifespan of telomere dysfunctional mice. Nat. Commun..

[bib90] Mohrin M., Bourke E., Alexander D., Warr M.R., Barry-Holson K., Le Beau M.M., Morrison C.G., Passegué E. (2010). Hematopoietic stem cell quiescence promotes error-prone DNA repair and mutagenesis. Cell Stem Cell.

[bib91] Morgan M.A., Shilatifard A. (2015). Chromatin signatures of cancer. Genes Dev..

[bib92] Norddahl G.L., Pronk C.J., Wahlestedt M., Sten G., Nygren J.M., Ugale A., Sigvardsson M., Bryder D. (2011). Accumulating mitochondrial DNA mutations drive premature hematopoietic aging phenotypes distinct from physiological stem cell aging. Cell Stem Cell.

[bib93] North B.J., Rosenberg M.A., Jeganathan K.B., Hafner A.V., Michan S., Dai J., Baker D.J., Cen Y., Wu L.E., Sauve A.A. (2014). SIRT2 induces the checkpoint kinase BubR1 to increase lifespan. EMBO J..

[bib94] O’Brien L.E., Soliman S.S., Li X., Bilder D. (2011). Altered modes of stem cell division drive adaptive intestinal growth. Cell.

[bib95] Ohm J.E., McGarvey K.M., Yu X., Cheng L., Schuebel K.E., Cope L., Mohammad H.P., Chen W., Daniel V.C., Yu W. (2007). A stem cell-like chromatin pattern may predispose tumor suppressor genes to DNA hypermethylation and heritable silencing. Nat. Genet..

[bib96] Passegué E., Wagers A.J., Giuriato S., Anderson W.C., Weissman I.L. (2005). Global analysis of proliferation and cell cycle gene expression in the regulation of hematopoietic stem and progenitor cell fates. J. Exp. Med..

[bib97] Passos J.F., Nelson G., Wang C., Richter T., Simillion C., Proctor C.J., Miwa S., Olijslagers S., Hallinan J., Wipat A. (2010). Feedback between p21 and reactive oxygen production is necessary for cell senescence. Mol. Syst. Biol..

[bib98] Pearson K.J., Lewis K.N., Price N.L., Chang J.W., Perez E., Cascajo M.V., Tamashiro K.L., Poosala S., Csiszar A., Ungvari Z. (2008). Nrf2 mediates cancer protection but not prolongevity induced by caloric restriction. Proc. Natl. Acad. Sci. USA.

[bib99] Rai T.S., Cole J.J., Nelson D.M., Dikovskaya D., Faller W.J., Vizioli M.G., Hewitt R.N., Anannya O., McBryan T., Manoharan I. (2014). HIRA orchestrates a dynamic chromatin landscape in senescence and is required for suppression of neoplasia. Genes Dev..

[bib100] Rakyan V.K., Down T.A., Maslau S., Andrew T., Yang T.P., Beyan H., Whittaker P., McCann O.T., Finer S., Valdes A.M. (2010). Human aging-associated DNA hypermethylation occurs preferentially at bivalent chromatin domains. Genome Res..

[bib101] Rando T.A. (2006). Stem cells, ageing and the quest for immortality. Nature.

[bib102] Rera M., Bahadorani S., Cho J., Koehler C.L., Ulgherait M., Hur J.H., Ansari W.S., Lo T., Jones D.L., Walker D.W. (2011). Modulation of longevity and tissue homeostasis by the Drosophila PGC-1 homolog. Cell Metab..

[bib103] Ricke R.M., van Ree J.H., van Deursen J.M. (2008). Whole chromosome instability and cancer: a complex relationship. Trends Genet..

[bib104] Risques R.A., Lai L.A., Brentnall T.A., Li L., Feng Z., Gallaher J., Mandelson M.T., Potter J.D., Bronner M.P., Rabinovitch P.S. (2008). Ulcerative colitis is a disease of accelerated colon aging: evidence from telomere attrition and DNA damage. Gastroenterology.

[bib105] Ritsma L., Ellenbroek S.I., Zomer A., Snippert H.J., de Sauvage F.J., Simons B.D., Clevers H., van Rheenen J. (2014). Intestinal crypt homeostasis revealed at single-stem-cell level by in vivo live imaging. Nature.

[bib106] Rossi D.J., Bryder D., Seita J., Nussenzweig A., Hoeijmakers J., Weissman I.L. (2007). Deficiencies in DNA damage repair limit the function of haematopoietic stem cells with age. Nature.

[bib107] Rudolph K.L., Chang S., Lee H.W., Blasco M., Gottlieb G.J., Greider C., DePinho R.A. (1999). Longevity, stress response, and cancer in aging telomerase-deficient mice. Cell.

[bib108] Rudolph K.L., Millard M., Bosenberg M.W., DePinho R.A. (2001). Telomere dysfunction and evolution of intestinal carcinoma in mice and humans. Nat. Genet..

[bib109] Sahin E., DePinho R.A. (2012). Axis of ageing: telomeres, p53 and mitochondria. Nat. Rev. Mol. Cell Biol..

[bib110] Schlesinger Y., Straussman R., Keshet I., Farkash S., Hecht M., Zimmerman J., Eden E., Yakhini Z., Ben-Shushan E., Reubinoff B.E. (2007). Polycomb-mediated methylation on Lys27 of histone H3 pre-marks genes for de novo methylation in cancer. Nat. Genet..

[bib111] Seluanov A., Mittelman D., Pereira-Smith O.M., Wilson J.H., Gorbunova V. (2004). DNA end joining becomes less efficient and more error-prone during cellular senescence. Proc. Natl. Acad. Sci. USA.

[bib112] Serrano M., Blasco M.A. (2007). Cancer and ageing: convergent and divergent mechanisms. Nat. Rev. Mol. Cell Biol..

[bib113] Shaver-Walker P.M., Urlando C., Tao K.S., Zhang X.B., Heddle J.A. (1995). Enhanced somatic mutation rates induced in stem cells of mice by low chronic exposure to ethylnitrosourea. Proc. Natl. Acad. Sci. USA.

[bib114] Shih A.H., Abdel-Wahab O., Patel J.P., Levine R.L. (2012). The role of mutations in epigenetic regulators in myeloid malignancies. Nat. Rev. Cancer.

[bib115] Shlush L.I., Zandi S., Mitchell A., Chen W.C., Brandwein J.M., Gupta V., Kennedy J.A., Schimmer A.D., Schuh A.C., Yee K.W., HALT Pan-Leukemia Gene Panel Consortium (2014). Identification of pre-leukaemic haematopoietic stem cells in acute leukaemia. Nature.

[bib116] Sirin O., Lukov G.L., Mao R., Conneely O.M., Goodell M.A. (2010). The orphan nuclear receptor Nurr1 restricts the proliferation of haematopoietic stem cells. Nat. Cell Biol..

[bib117] Smith B.D., Smith G.L., Hurria A., Hortobagyi G.N., Buchholz T.A. (2009). Future of cancer incidence in the United States: burdens upon an aging, changing nation. J. Clin. Oncol..

[bib118] Snippert H.J., van der Flier L.G., Sato T., van Es J.H., van den Born M., Kroon-Veenboer C., Barker N., Klein A.M., van Rheenen J., Simons B.D., Clevers H. (2010). Intestinal crypt homeostasis results from neutral competition between symmetrically dividing Lgr5 stem cells. Cell.

[bib119] Sousa-Victor P., Gutarra S., García-Prat L., Rodriguez-Ubreva J., Ortet L., Ruiz-Bonilla V., Jardí M., Ballestar E., González S., Serrano A.L. (2014). Geriatric muscle stem cells switch reversible quiescence into senescence. Nature.

[bib120] Sun D., Luo M., Jeong M., Rodriguez B., Xia Z., Hannah R., Wang H., Le T., Faull K.F., Chen R. (2014). Epigenomic profiling of young and aged HSCs reveals concerted changes during aging that reinforce self-renewal. Cell Stem Cell.

[bib121] Sun J., Ramos A., Chapman B., Johnnidis J.B., Le L., Ho Y.J., Klein A., Hofmann O., Camargo F.D. (2014). Clonal dynamics of native haematopoiesis. Nature.

[bib122] Swierczek S.I., Agarwal N., Nussenzveig R.H., Rothstein G., Wilson A., Artz A., Prchal J.T. (2008). Hematopoiesis is not clonal in healthy elderly women. Blood.

[bib123] Tao K.S., Urlando C., Heddle J.A. (1993). Comparison of somatic mutation in a transgenic versus host locus. Proc. Natl. Acad. Sci. USA.

[bib124] Tao S., Tang D., Morita Y., Sperka T., Omrani O., Lechel A., Sakk V., Kraus J., Kestler H.A., Kühl M., Rudolph K.L. (2015). Wnt activity and basal niche position sensitize intestinal stem and progenitor cells to DNA damage. EMBO J..

[bib125] Teschendorff A.E., Menon U., Gentry-Maharaj A., Ramus S.J., Weisenberger D.J., Shen H., Campan M., Noushmehr H., Bell C.G., Maxwell A.P. (2010). Age-dependent DNA methylation of genes that are suppressed in stem cells is a hallmark of cancer. Genome Res..

[bib126] Tomasetti C., Vogelstein B. (2015). Cancer etiology. Variation in cancer risk among tissues can be explained by the number of stem cell divisions. Science.

[bib127] Townsley D.M., Dumitriu B., Young N.S. (2014). Bone marrow failure and the telomeropathies. Blood.

[bib128] Trifunovic A., Wredenberg A., Falkenberg M., Spelbrink J.N., Rovio A.T., Bruder C.E., Bohlooly-Y M., Gidlöf S., Oldfors A., Wibom R. (2004). Premature ageing in mice expressing defective mitochondrial DNA polymerase. Nature.

[bib129] Tsai J.J., Dudakov J.A., Takahashi K., Shieh J.H., Velardi E., Holland A.M., Singer N.V., West M.L., Smith O.M., Young L.F. (2013). Nrf2 regulates haematopoietic stem cell function. Nat. Cell Biol..

[bib130] Vas V., Senger K., Dörr K., Niebel A., Geiger H. (2012). Aging of the microenvironment influences clonality in hematopoiesis. PLoS ONE.

[bib131] Vaziri H., Dragowska W., Allsopp R.C., Thomas T.E., Harley C.B., Lansdorp P.M. (1994). Evidence for a mitotic clock in human hematopoietic stem cells: loss of telomeric DNA with age. Proc. Natl. Acad. Sci. USA.

[bib132] Velarde M.C., Demaria M., Campisi J. (2013). Senescent cells and their secretory phenotype as targets for cancer therapy. Interdiscip. Top. Gerontol..

[bib133] Vermeulen L., Morrissey E., van der Heijden M., Nicholson A.M., Sottoriva A., Buczacki S., Kemp R., Tavaré S., Winton D.J. (2013). Defining stem cell dynamics in models of intestinal tumor initiation. Science.

[bib134] Walkley C.R., Fero M.L., Chien W.M., Purton L.E., McArthur G.A. (2005). Negative cell-cycle regulators cooperatively control self-renewal and differentiation of haematopoietic stem cells. Nat. Cell Biol..

[bib135] Walter D., Lier A., Geiselhart A., Thalheimer F.B., Huntscha S., Sobotta M.C., Moehrle B., Brocks D., Bayindir I., Kaschutnig P. (2015). Exit from dormancy provokes DNA-damage-induced attrition in haematopoietic stem cells. Nature.

[bib136] Wang J., Sun Q., Morita Y., Jiang H., Gross A., Lechel A., Hildner K., Guachalla L.M., Gompf A., Hartmann D. (2012). A differentiation checkpoint limits hematopoietic stem cell self-renewal in response to DNA damage. Cell.

[bib137] Wang J., Lu X., Sakk V., Klein C.A., Rudolph K.L. (2014). Senescence and apoptosis block hematopoietic activation of quiescent hematopoietic stem cells with short telomeres. Blood.

[bib138] Warr M.R., Binnewies M., Flach J., Reynaud D., Garg T., Malhotra R., Debnath J., Passegué E. (2013). FOXO3A directs a protective autophagy program in haematopoietic stem cells. Nature.

[bib139] Weidner C.I., Lin Q., Koch C.M., Eisele L., Beier F., Ziegler P., Bauerschlag D.O., Jöckel K.H., Erbel R., Mühleisen T.W. (2014). Aging of blood can be tracked by DNA methylation changes at just three CpG sites. Genome Biol..

[bib140] Widschwendter M., Fiegl H., Egle D., Mueller-Holzner E., Spizzo G., Marth C., Weisenberger D.J., Campan M., Young J., Jacobs I., Laird P.W. (2007). Epigenetic stem cell signature in cancer. Nat. Genet..

[bib141] Willcox B.J., Donlon T.A., He Q., Chen R., Grove J.S., Yano K., Masaki K.H., Willcox D.C., Rodriguez B., Curb J.D. (2008). FOXO3A genotype is strongly associated with human longevity. Proc. Natl. Acad. Sci. USA.

[bib142] Williams G.C. (1957). Pleiotropy, natural selection and the evolution of senescence. Evolution.

[bib143] Wilson A., Laurenti E., Oser G., van der Wath R.C., Blanco-Bose W., Jaworski M., Offner S., Dunant C.F., Eshkind L., Bockamp E. (2008). Hematopoietic stem cells reversibly switch from dormancy to self-renewal during homeostasis and repair. Cell.

[bib144] Winton D.J., Blount M.A., Ponder B.A. (1988). A clonal marker induced by mutation in mouse intestinal epithelium. Nature.

[bib145] Xie M., Lu C., Wang J., McLellan M.D., Johnson K.J., Wendl M.C., McMichael J.F., Schmidt H.K., Yellapantula V., Miller C.A. (2014). Age-related mutations associated with clonal hematopoietic expansion and malignancies. Nat. Med..

[bib146] Yancik R. (2005). Population aging and cancer: a cross-national concern. Cancer J..

[bib147] Ye M., Zhang H., Amabile G., Yang H., Staber P.B., Zhang P., Levantini E., Alberich-Jordà M., Zhang J., Kawasaki A., Tenen D.G. (2013). C/EBPa controls acquisition and maintenance of adult haematopoietic stem cell quiescence. Nat. Cell Biol..

[bib148] Yilmaz O.H., Katajisto P., Lamming D.W., Gültekin Y., Bauer-Rowe K.E., Sengupta S., Birsoy K., Dursun A., Yilmaz V.O., Selig M. (2012). mTORC1 in the Paneth cell niche couples intestinal stem-cell function to calorie intake. Nature.

